# Family structures and parents’ occupational models: its impact on children’s diabetes

**DOI:** 10.1007/s00592-023-02187-9

**Published:** 2023-10-17

**Authors:** Pauline Schlarb, Janina M. Büttner, Sascha R. Tittel, Kirsten Mönkemöller, Esther Müller-Godeffroy, Claudia Boettcher, Angela Galler, Gabriele Berger, Burkhard Brosig, Reinhard W. Holl

**Affiliations:** 1 Centre of Child and Adolescent Medicine, Division of Family- and Child-Psychosomatics, Justus Liebig University, Klinikstrasse 36, 35392 Giessen, Germany; 2 ZIBMT, Institute of Epidemiology and Medical Biometry, University of Ulm, Albert Einstein Alle 41, 89075 Ulm, Germany; 3 German Centre for Diabetes Research (DZD), Ingolstädter Landstrasse, 185764 Munich-Neuherberg, Germany; 4 Department of Pediatric and Adolescent Medicine, Kliniken Der Stadt Köln gGmbH, Amsterdamer Strasse 59, 50735 Cologne, Germany; 5 Department of Pediatric and Adolescent Medicine, University of Luebeck, Ratzeburger Allee 160, 23538 Luebeck, Germany; 6 Pediatric Endocrinology and Diabetology, University of Berne, University Children’s Hospital, Freiburgstrasse 15, 3010 Berne, Switzerland; 7 Charité - Universitätsmedizin Berlin, Corporate Member of Freie Universität Berlin and Humboldt-Universität Zu Berlin, Sozialpädiatrisches Zentrum, Augustenburger Platz 1, 13353 Berlin, Germany; 8 Department of Paediatrics and Adolescent Medicine, Medical University of Vienna, Waehringer Guertel 18-20, 1090 Vienna, Austria; 9 Pediatric Diabetes Outpatient Clinic, Health Care Centre Vienna Floridsdorf, Karl-Aschenbrenner-Gasse 3, 1210 Vienna, Austria

**Keywords:** T1DM, family structure, parents’ working time models, German Diabetes Prospective Follow-up Registry (DPV), family psychosomatics, adherence

## Abstract

**Aims:**

This study examines how family-related factors influence the management of children and adolescents with type 1 diabetes (T1DM). We investigate the relationship between family patterns, parental work schedules and metabolic control.

**Materials and methods:**

We analysed data from a nationwide diabetes survey (DPV) focusing on HbA1c, severe hypoglycaemia, diabetic ketoacidosis, hospital admissions and inpatient treatment duration. We used linear regression and negative binomial regression models. Our study includes 15,340 children under the age of 18 with data on family structure and parental division of labour.

**Results:**

Children from two-parent households have better HbA_1c_ outcomes than children from single-parent, blended or no-parent households (*p* < .0001). Higher HbA_1C_ levels are associated with children living with an unemployed father, as opposed to those with full-time working parents or with a full-time working father and a part-time working mother (*p* < .001).

**Conclusions:**

These findings emphasise the importance of carefully considering family structure and working time models in the management of paediatric T1DM. Our results highlight risk factors within the family environment and emphasise the need for family-focused counselling of high-risk patients or severe cases in clinical practice.

## Introduction

Type 1 diabetes mellitus (T1DM) as a chronic metabolic disease with an autoimmune aetiology and an increasing incidence poses a challenge for the continuous monitoring of patients’ health status to ensure a positive long-term prognosis. In this paper, we study the interrelation of family forms and division of labour between caregivers and the corresponding metabolic adjustment for T1DM-affected children and adolescents. Socioeconomic status and family functionality play an essential role in the effectiveness of T1DM-treatment [1]. Thus, psychosocial factors should be considered right from the start and during the entire treatment of T1DM, as they are known prognostic factors for the maintenance of physical health [2].

From a perspective of family sociology, family arrangements and lifestyles have changed profoundly over the last decades; modern families are gaining more structural complexity due to frequent separations, divorces and re-marriages. Hence, the proportion of new family constellations like one biological parent with their partner, one-parent families with single mothers, and rainbow-families has increased [3]. Baechle et al. [4] described ‘non-classical’ family structures being associated with substantially poorer outcomes for T1DM-related treatment parameters. Furthermore, adolescents living with one parent and having a low socioeconomic status (SES) are more susceptible to a reduced 'health-related quality of life' [5]. An Italian study ascertained a correlation between living with one parent and worse glycaemic control in children with T1DM [6].

As well as contemporary family models, women’s emancipation and double career projects in couples have also made a difference to family functioning. The ‘traditional breadwinner-homemaker arrangement’ has become rarer [7]. It is known from Norway that living in families with both biological parents and a high SES (including education and employment) is associated with better T1DM parameters [8].

Based on these findings, this study analyses the interrelation between family structure and working time models of both parents or workload of a single parent. We hypothesize that the psychosocial risk of living in a one-parent household results in higher HbA_1c_ parameters, higher rates of severe hypoglycaemia (SH), diabetic ketoacidosis (DKA) and longer inpatient treatment. Conversely, a full-time working father in a two-parents household is expected to predict a lower HbA_1c_, lower rates of hospital admission, SH and DKA.

## Materials and methods

### Data and participants

The current study is based on data from the multicentre German, Austrian, Swiss, and Luxembourgian Diabetes Prospective Follow-up Registry (i.e., Diabetes-Patienten-Verlaufsdokumentation [DPV]) [9]. This study, ‘Family structure and adherence of type I diabetes mellitus’, received approval from the Ethics Committee in October 2020 (Vote #185/20, department of medicine Justus-Liebig University Gießen). As of August 2023, 490 diabetes centres (hospitals and outpatient centres) and 601 200 patients with diabetes are included in the DPV registry. The data are pseudonymized and transmitted for central plausibility checks and analyses to the Institute of Epidemiology and Medical Biometry of Ulm University (Ulm, Germany). After reporting inconsistent data back to participating centres for validation and correction, the data are then completely anonymised prior to analysis.

**Fig. 1:**
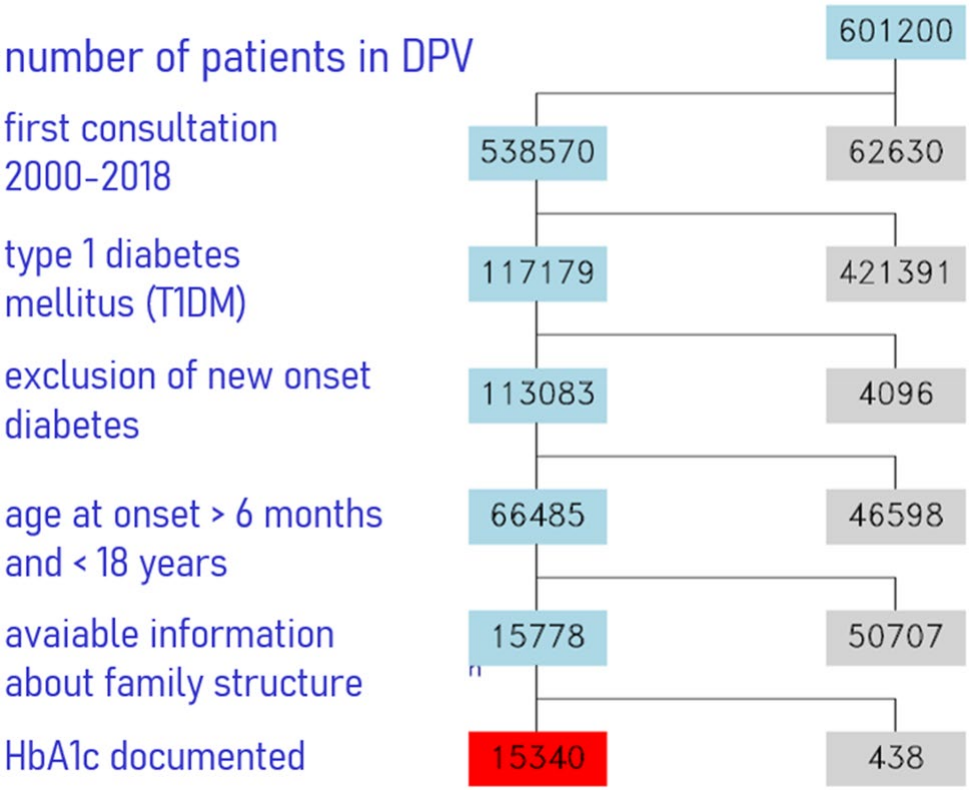
Selection of the Study Population in DPV (Diabetes Patient Follow-up Registry) (grey: excluded, blue: retained, red: final collective of patients) (color figure online)

Out of the DPV registry, patients who first presented with diabetes before 2000 or after 2018 were excluded (Fig. 1). We included patients with a clinical diagnosis of T1DM between six months and 18 years at the time of diagnosis with information on family structure, parents’ employment characteristics and HbA_1c_. We included the data of 15,340 patients from 301 diabetes centres in our study sample (286 German centres, 14 Austrian centres, 1 Luxembourgian centre). The data pool was aggregated for a 2-year period prior and following the last psychosocial data collection.

### Variables

Demographic data included age, age at diabetes onset, duration of diabetes, sex, and migratory background (patient or at least one parent born outside Germany, Austria, Switzerland, or Luxembourg).

Clinical parameters were evaluated 2 years prior and following the last psychosocial entry. They included body-mass index standard deviation score (BMI SDS), the daily dose of insulin (IE/kg), the use of continuous or flash glucose monitoring, the type of insulin therapy (multiple daily injections or insulin pump treatment) and HbA_1c_ (percentage; mmol/mol). Further outcome measures included rates of severe hypoglycaemia (with or without coma) and diabetic ketoacidosis, rates of diabetes-associated hospital admissions (i.e., hospitalization), and inpatient days in hospital each year due to diabetes. BMI SDS was calculated using the national KiGGS (German Health Interview and Examination Survey for Children and Adolescents) reference data in Germany [10]. HbA_1c_ values were measured locally and standardized mathematically to the Diabetes Control and Complications Trial (DCCT) reference range (4.05–6.05%) using the multiple of the mean (MOM) method [11]. Severe hypoglycaemia (SH)—an event requiring external assistance by another person; hypoglycaemic coma (SHC)—severe hypoglycaemia associated with seizure or loss of consciousness; and an event of diabetic ketoacidosis (DKA)—a pH of less than 7.3 or serum bicarbonate of less than 15 mmol/L or both were defined according to the guidelines of International Society for Pediatric and Adolescent Diabetes (ISPAD) [12, 13]. Rates of severe hypoglycaemia, DKA, and hospital admission rates were presented as events per 100 patient years. Length of inpatient treatment was given as the number of inpatient days per patient and year.

**Fig. 2:**
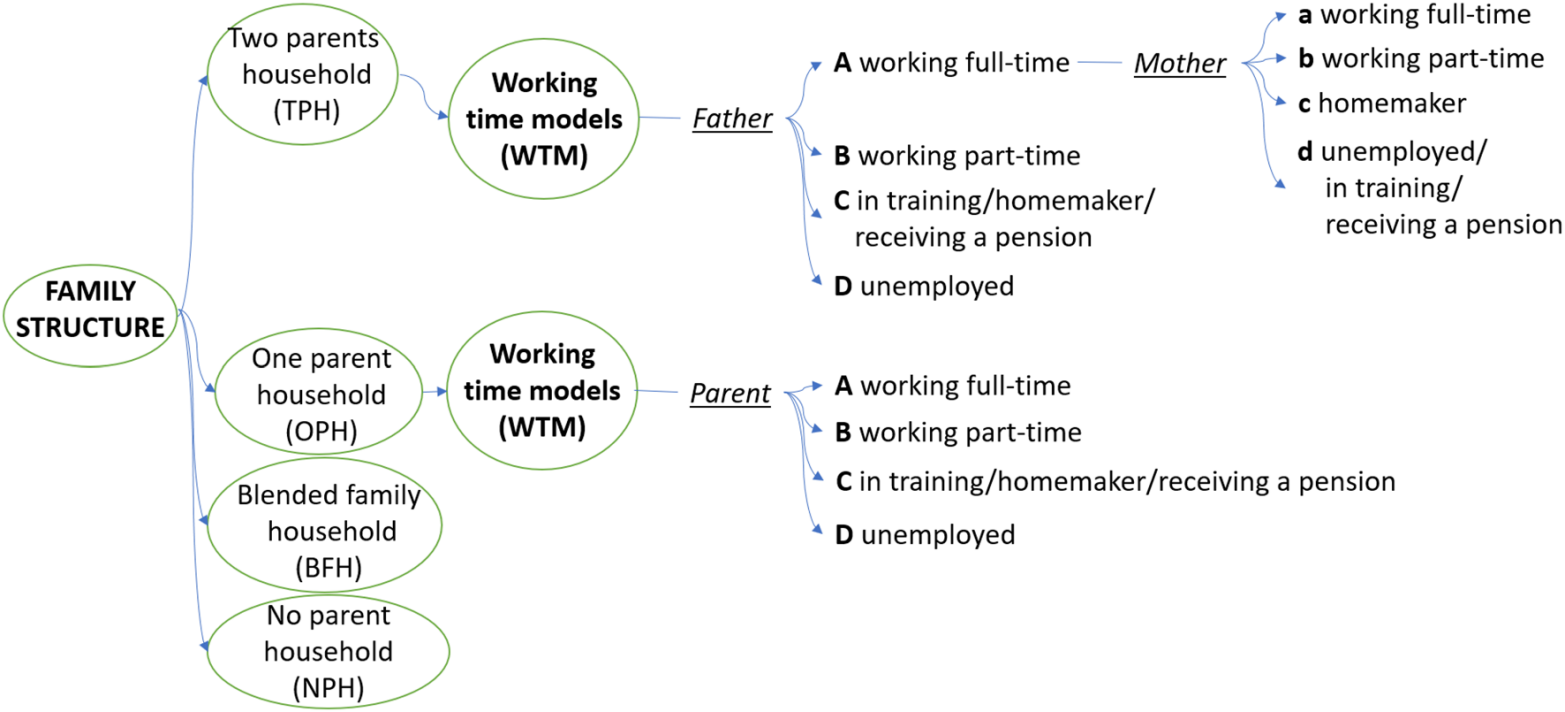
Categorial system applied for the variables family structure and working time models

The database includes information on family structure and working time models (WTM) of the children’s caretakers and was stratified according to these categories. We defined living with both biological parents as a two-parents household (TPH) and living with one biological parent as a one-parent household (OPH). Blended family households (BFH) were defined as living with one biological parent and his/her partner in the household. Other living conditions (e.g., living with other relatives, adoptive parents, boarding school, foster care homes of the child welfare service, etc.) were categorized as living without a biological parent (no parent household, NPH). Working time models differentiate into working full-time (A), part-time (B), homemaker, being in training, receiving a pension (C) and being unemployed (D). The categorical system focuses on the father regarding two parent households and the mother in terms of one-parent households (Fig. 2).

### Statistical analysis

The data evaluation and statistical analysis was performed using SAS 9.4 (SAS Institute, Cary, NC, USA). Linear regression models were applied to calculate and compare HbA_1c_ (% or mmol/mol) between family structures and working time models. Rates of SH, SHC, DKA, and hospital admission rates and length of inpatient treatment were analysed via negative binomial regression using individual time under risk as offset. All models were adjusted for age group (< 10, 10 to 15, ≥ 15 years), sex, diabetes duration group (< 2, 2 to 5, ≥ 5 years) and migratory background (yes, no). Results are presented as least-square means with 95% confidence intervals. The Tukey–Kramer method was used for multiple testing in unbalanced data. Goodness-of-fit was assessed via R-Squared. Models were tested for each dependent variable using analysis of variance.

## Results

The results are presented by the categories *family structure* and *working time models*. The categories will be referred to as depicted in Fig. 2.

Out of the entire cohort 52.0% were males and migratory background was present in 22.6% of the patients. The median age at diagnosis of T1DM was 8.1 years (Q1–Q3: 4.7–11.4) and 12.8 years (9.3–15.4) during follow-up. More than 70% (10,790) of subjects lived in a two-parents household, 17.8% (2726) in a one-parent household, 8.5% (1296) lived in blended families and 3.4% (528) without a biological parent. All *F*-tests for the analyses of variance were significant (*p* < 0.01) in all models tested.

### Family structure

Patients living in a two-parents household had significantly lower HbA_1c_ levels compared to other living conditions (7.70; 60.70 vs. 8.06; 64.63 for OPHs; 8.07; 64.66 for BFHs; 8.21; 66.22 for NPHs) as well as lower rates of DKA (1.79) than subjects living in one-parent households (3.41), blended family-households (4.00), and no parent households (7.19) (see Table 1).

**Table 1:** Family structure, working time models, and quality of diabetes care

Family structure	Form of social status	Categories	Frequency	HbA1c^1^	Severe hypoglycaemia (SH)^1^	Rate of SH with coma (SHC)^1^	Diabetic ketoacidosis (DKA)^1^	Length of hospital stay^1^	Hospital admission rate^1^
				(mmol/mol)/(%)	(per 100 patient years)	(per 100 patient years)	(per 100 patient years)	(days per patient year)	(per 100 patient years)
Two-parents household (TPH)^2^			**10**,**790**	**60**.70*** (60.43; 60.96)	**14**.**86**	**3**.15**	**1**.79***	**12**.82***	**57**.46***
				**7**.70*** (7.68; 7.73)	(13.99; 15.78)	(2.88; 3.43)	(1.62; 1.99)	(12.51; 13.12)	(56.31; 58.65)
	Working time models (WTM)^3^	***Aa**** Father working full-time—mother working full-time*	1848	**59**.85*** (59.24; 60.46)	**13**.**26**	**2**.**75**	**1**.**64**	**15**.41***	**60**.58**
				**7**.63*** (7.57; 7.68)	(11.35; 15.49)	(2.20; 3.44)	(1.26; 2.13)	(14.56; 16.32)	(57.70; 63.60)
		***Ab**** Father working full-time—mother working part-time*	4446	**59**.71*** (59.32; 60.10)	**12**.**95**	**2**.48*	**1**.53*	**11**.97***	**53**.66***
				**7**.61*** (7.58; 7.65)	(11.74; 14.29)	(2.14; 2.88)	(1.28; 1.82)	(11.53; 12.42)	(51.95; 55.42)
		***Ac**** Father working full-time—mother homemaker*	2339	**60**.60** (60.06; 61.14)	**15**.**88**	**3**.**09**	**1**.**86**	**12**.28***	**55**.64***
				**7**.69** (7.65; 7.74)	(13.91; 18.14)	(2.54; 3.76)	(1.48; 2.36)	(11.66; 12.93)	(53.21; 58.18)
		***Ad**** father working full-time—mother is unemployed/in training/receiving a pension*	240	**60**.**91** (59.34; 62.38)	**10**.**13**	**2**.**9**	**1**.**46**	**14**.**84**	**66**.29*
				**7**.**72** (7.57; 7.88)	(6.43; 15.94)	(1.52; 5.52)	(0.68; 3.14)	(12.78; 17.24)	(58.10; 75.64)
		***B**** Father working part-time*	291	**60**.**87** (59.34; 62.38)	**17**.**76**	**2**.**98**	**1**.**79**	**12**.14*	**62**.**22**
				**7**.**72** (7.58; 7.86)	(12.33; 25.57)	(1.75; 5.08)	(0.98; 3.24)	(10.60; 13.91)	(55.29; 70.03)
		***C**** Father in training/homemaker/receiving a pension*	302	**62**.**53** (61.04; 64.03)	**17**.**16**	**4**.**32**	**2**.**41**	**14**.73***	**75**.76***
				**7**.**87** (7.74; 8.01)	(11.95; 24.64)	(2.67; 6.99)	(1.39; 4.21)	(12.90; 16.82)	(67.69; 84.79)
		***D**** Father is unemployed*	369	**63**.53** (62.17; 64.89)	**21**.**09**	**5**.12*	**2**.96*	**16**.59***	**72**.16***
				**7**.96** (7.84; 8.09)	(15.36; 28.97	(3.34; 7.85)	(1.84; 4.74)	(14.66; 18.77)	(64.97; 80.15)
One-parent household (OPH)^2^			**2726**	**64**.63*** (64.10; 65.16)	**15**.**88**	**3**.68*	**3**.41***	**16**.07***	**76**.02***
				**8**.06*** (8.02; 8.11)	(14.11; 17.87)	(3.11; 4.35)	(2.91; 3.99)	(15.38; 16.79)	(73.25; 78.89)
	Working time models (WTM)^3^	***A**** Parent working full-time*	935	**64**.**73** (63.76; 65.70)	**16**.**21**	**3**.**12**	**3**.**26**	**16**.**19**	**72**.77*
				**8**.**07** (7.98; 8.16)	(13.23; 19.86)	(2.33; 4.18)	(2.49; 4.28)	(15.05; 17.42)	(68.36; 77.45)
		***B**** Parent working part-time*	931	**64**.**78** (63.82; 65.74)	**16**.**67**	**3**.**96**	**3**.**34**	**14**.63*	**72**.25*
				**8**.**08** (7.99; 8.17)	(13.70; 20.29)	(3.04; 5.16)	(2.56; 4.35)	(13.60; 15.74)	(67.91; 76.86)
		***C**** Parent in training/homemaker/receiving a pension*	428	**66**.**02** (64.57; 67.47)	**14**.**93**	**4**.**56**	**3**.**91**	**16.36**	**84**.50*
				**8**.**19** (8.06; 8.32)	(11.04; 20.20)	(3.09; 6.71)	(2.69; 5.69)	(14.70; 18.20)	(77.31; 92.36)
		***D**** Parent unemployed*	243	**67**.**10** (65.21; 68.99)	**13**.**17**	**2**.**9**	**3**.**39**	**18**.36*	**84**.**53**
				**8**.**29** (8.12; 8.46)	(8.70; 19.93)	(1.61; 5.21)	(2.00; 5.76)	(15.96; 21.12)	(75.07; 95.19)
Blended Family household (BFH)^2^			**1296**	**64**.65*** (63.89; 65.42)	**16**.**04**	**4**.36*	**3**.97***	**16**.10***	**80**.69***
				**8**.07*** (8.00; 8.13)	(13.52; 19.02)	(3.47; 5.48)	(3.20; 4.93)	(15.12; 17.15)	(76.53; 85.08)
No parent household (NPH)^2^			**528**	**66**.22*** (65.02; 67,43)	**20**.**641**	**6**.29**	**7**.19***	**19**.01***	**106**.29***
				**8**.21*** (8.10; 8.32)	(15.72; 26.51)	(4.51; 8.79)	(5.42; 9.54)	(17.26; 20.94)	(98.23; 115.01)

^1^Data expressed as mean (lower vs. upper bound of 95%-confidence interval); least square means adjusted for age, duration of diabetes, sex and a background of migration

^2^Significant results compare different family structures

^3^Significant results compare different working time models

***significant for *p* < .0001

**significant for *p* < .01

*significant for *p* < .05

The rates of hypoglycaemic coma were higher for children living in no-parent households (6.29), compared with children living in two-parent households (3.15), single-parent households (3.68) and blended family households (4.36). Rates of severe hypoglycaemia did not differ significantly between these categories. Length of inpatient treatment and hospital admission rates were lowest for adolescents living in a two-parent household (12.82; 57.46 vs. 16.07; 76.02 for OPHs; 16.10; 80.69 for BFHs; 19.01; 106.29 for NPHs). Table 2 compares the effects between two-parent and single-parent households, with overall poorer outcomes for children living in two-parent households.

**Table 2:** Effect of family stru﻿cture

	(A) Two-parents household (TPH)	(B) One-parent household (OPH)	adj p*
HbA_1c_^a^ (%)	7.7	8.06	< 0.0001^1^
	(7.68; 7.73)	(8.02; 8.11)	
HbA_1c_^a^ (mmol/L)	60.69	64.63	< 0.0001^1^
	(60.43; 60.96)	(64.10; 65.16)	
Rate of severe hypoglycaemia^a^	14.86	15.88	0.0935^2^
(per 100 patient-years)	(13.99; 15.78)	(14.11; 17.87)	
Rate of hypoglycaemia with coma^a^	3.15	3.68	0.0005^2^
(per 100 patient-years)	(2.88; 3.43)	(3.11; 4.35)	
Rate of episodes with DKA^a^	1.79	3.41	< 0.0001^2^
(per 100 patient-years)	(1.62; 1.99)	(2.91; 3.99)	
Length of hospital stay^a^	12.82	16.07	< 0.0001^2^
(days per patient-year)	(12.51; 13.12)	(15.38; 16.79)	
Hospital admission rate^a^	57.46	76.02	< 0.0001^2^
(per 100 patient-years)	(56.31; 58.65)	(73.25; 78.89)	

^a^Data expressed as mean (95% confidence interval)

^1^linear regression model

^2^negative binomial regression model; least square means adjusted for age, duration of diabetes, sex, and a background of migration

Goodness-of-fit analysis showed 14% of the variability concerning the HbA_1C_ parameters can be explained by family structure (*R*^2^ = 0.14, *F*_(3, 15,330)_ = 92.38, *p* < 0.0001) (see Table 4).

### Working time models: two-parents household (TPH, WTM, Aa-D)

The adjusted outcomes indicated significantly higher HbA_1c_ parameters in children of parents with working time models of category C (7.88; 62.58) and D (7.97; 63.55) versus categories Aa (7.62; 59.82) and Ab (7.61; 59.71) as well as category C vs category Ac (7.70; 60.60). Lower rates of hypoglycaemic coma were recorded in children from couples of category Ab compared to D (2.48 vs. 5.12). Rates of severe hypoglycaemia and DKA did not differ significantly between working time models (Table 1). In group comparison, the length of inpatient treatment differed between categories with longer inpatient treatment for C (14.73), D (16.60), Aa (15.41) and shorter treatment for Ab (11.97), Ac (12.28) and B (12.14). Higher hospital admission rates were reported for categories C (75.76) and D (72.16) as well as for Aa versus Ab (60.58 vs. 53.66) and Ab vs Ad (53.66 vs. 66.29). Table 3 contrasts the findings for the categories Ab and D regarding the working time models. Living with a full-time working father and a part-time working mother came with overall lower outcomes compared to living with an unemployed father.

**Table 3:** Effect of working time models (TPH)

	(Ab) Father working full-time—mother working part-time	(D) Father is unemployed	adj p*
HbA_1c_^a^ (%)	7.611	7.96	< 0.0001^1^
	(7.58; 7.65)	(7.84; 8.09)	
HbA_1c_^a^ (mmol/L)	59.71	63.53	< 0.0001^1^
	(59.32; 60.10)	(62.17; 64.89)	
Rate of severe hypoglycaemia^a^	12.95	21.09	0.0621^1^
(per 100 patient-years)	(11.74; 14.29)	(15.36; 28.97)	
Rate of hypoglycaemia with coma^a^	2.48	5.12	0.0280^2^
(per 100 patient-years)	(2.14; 2.88)	(3.34; 7.85)	
Rate of episodes with DKA^a^	1.53	2.96	0.0100^2^
(per 100 patient-years)	(1.28; 1.82)	(1.84; 4.74)	
Length of hospital stay^a^	11.97	16.59	< 0.0001^2^
(days per patient-year)	(11.53; 12.42)	(14.66; 18.77)	
Hospital admission rate^a^	53.66	72.16	< 0.0001^2^
(per 100 patient-years)	(51.95; 55.42)	(64.97; 80.15)	

^a^Data expressed as mean (95% confidence interval)

^1^linear regression model

^2^negative binomial regression model; least square means adjusted for age, duration of diabetes, sex, and a background of migration

Goodness-of-fit analysis indicated 13% of the variability concerning the HbA_1C_ parameters can be explained by working time models in two-parent households (*R*^2^ = 0.13, *F*_(6, 9842)_ = 7.01, *p* < 0.0001) (see Table 4).

**Table 4:** Goodness of fit family models by dependent variables

	Family structure				Working time models (WTM)—Two-parents household (TPH)				Working time models (WTM)—One-parent household (OPH)			
	*R*^2^	*F*	*df*	*p*	*R*^2^	*F*	*df*	*p*	*R*^2^	*F*	*df*	*p*
HbA1c	0.141	92.38	3	< 0.0001^1^	0.132	7.01	6	< 0.0001^1^	0.103	2.2	3	0.0858^1^
			155,330				9842				2527	
Severe hypoglycaemia (SH)	0.006	2.07	3	< 0.01019^2^	0.007	2.84	6	0.0092^2^	0.009	0.4	3	0.7532^2^
			15,329				9841				2527	
Rate of SH with coma (SHC)	0.007	7.05	3	< 0.0001^2^	0.007	2.34	6	0.0290^2^	0.01	1.11	3	0.3445^2^
			15,329				9841				2527	
Diabetic ketoacidosis (DKA)	0.019	43.08	3	< 0.0001^2^	0.008	150	6	0.1723^2^	0.018	0.22	3	0.8841^2^
			15,329				9841				2527	
Length of hospital stay	0.026	48.99	3	< 0.0001^2^	0.021	13.44	6	< 0.0001^2^	0.021	3.22	3	0.0220^2^
			10,731				6609				1916	
Hospital admission rate	0.067	138.69	3	< 0.0001^2^	0.052	11.9	6	< 0.0001^2^	0.062	4.25	3	0.0053^2^
			15,329				9841				2527	

Goodness of fit via *R*^2^ and Type III Tests of Fixed Effects ^1^linear regression model

^2^negative binomial regression model

### Working time models: one-parent household (OS, OPH, A-D)

According to goodness-of-fit analysis, there was no additional significant information gained regarding the working time models in one-parent households for HbA_1c_ levels, DKA, severe hypoglycaemia and hypoglycaemic coma. Hospital admission rates were higher for category C (84.50) compared to A (72.77) and B (72.25). Length of inpatient treatment differed between category B (14.63) and D (18.36) (see Table 4).

## Discussion

Our study shows significant influences of family structure and working time models in two-parent households on children’s diabetes management. Better metabolic adjustment is seen in children from two-parent households compared to other living arrangements. In detail, the following points should be emphasised.

The data in Tables 1 and 2 depict significant differences in the key parameters of metabolic control comparing children in various family constellations. Living in a one-parent household is a clear risk factor for poor metabolic control in T1DM. These children have a significantly higher risk of metabolic derailment and therefore a higher risk of long-term complications. Conversely, living with both biological parents is a protective factor for diabetes control during course of disease.

“Modern” parents have more freedom and develop a variety of options for how they want to live with their children. This presents new challenges and can lead to insecurity and symptom burden in children [7]. Growing up without both biological parents increases the probability for children to have chronic diseases and problems with emotions or behaviour [14]. Children living with divorced parents appear to experience more overall stress due to the various aspects of separation [15]. Families with high levels of general stress should be identified as they are at risk of poorer health for both parents and children [16]. Consequently, the quality of life in children with T1DM is negatively correlated with family conflict, particularly conflict related to diabetes management [17]. These negative interactions with metabolic control are confirmed by our results—children from one-parent families, blended families and children living without biological parents have worse metabolic outcomes compared to children and adolescents from two-parent households. Similar results were reported by Baechle et al. [4]. About 15% of the variance in HbA_1c_ parameters can be explained by family structure and working time models. This underlines the effect of these psychosocial influences on diabetes adherence when all other possible influences are taken into account.

The data in Table 3 show differences in metabolic control comparing working time models. Paternal unemployment is a significant risk factor for poor diabetes outcomes in the child. There is a higher risk of life-threatening metabolic derailment and long-term consequences. Working parents are therefore a protective factor for metabolic control.

Parental care and involvement are necessary for positive living with T1DM [17, 18]. The risk of unemployment and low SES—which we found to be negatively associated with diabetes outcomes—predict mood disorders in other studies, and in general, parental emotional stress has a negative effect on children's metabolic control [19–21].

Family support may be helpful in preventing complications of T1DM [22]. Other studies have found an association between poorer diabetes control and a higher percentage of one-parent families, which could be due to either low SES or inadequate ability to provide adequate care for children [23].

Although family structure and work schedules play an important role in diabetes management, the pathway to family-oriented interventional improvement is largely unclear. The effect of family therapy is well documented at the level of individual cases [24]. At the level of meta-analyses, the effect of these approaches remains disappointing [25, 26].

The results of the study suggest aspects that could be considered in the management of chronic diseases in general and may provide guidance for other populations. Diabetes is a good paradigm for any chronic disease. The need for 24-h care and the possibility of an objective measure of treatment success, such as HbA_1c_, make diabetes suitable for such studies. In addition, the DPV registry is an enormously large database, including more than half of the children with T1DM in Germany [27]. Thus, this study may provide evidence on the influence of family structure on the overall care of chronically ill children. Possible new research approaches could include studying bronchial asthma and inflammatory bowel disease in relation to family structure.

### Strengths and limitations of the study

An obvious strength of the study is the large sample size. To date, most studies included less participants and were conducted in a single country or culture [8, 22, 23, 28]. This study combines data from three different countries, the data collection is well established, and the database has proven to be a matrix of intensive publication activity.

The interpretation of R^2^ can be limited so we complemented analyses of variance for all tested models. Nevertheless, we did not include further validation techniques like residual analysis to obtain an even more thorough understanding of the model’s performance.

The study is limited to the categorical system given in the data mask. Overall, the chosen classification reflects a family-related aspect of social status and can be associated with parental education, working hours, occupational status and household income [14]. The influence of parental income and education should be investigated in future research. The data were limited by the information provided and we had to exclude patients who did not provide information on family structure. In addition, future studies should include more diverse modern family forms (e.g., different forms of blended or rainbow families) in their research. This could be helpful in describing coping potential and identifying factors of clinical importance for better treatment options.

## Conclusion

The results of this study highlight the importance of carefully considering family structure, parental employment status and work schedules in the management of paediatric T1DM. Our findings highlight risk factors within the family environment and the need for family-focused counselling for high-risk patients or severe cases in clinical practice.

- Different family models may influence the quality of diabetes management at home.
- Living with both biological parents may be a protective factor.
- Single-parent families are particularly at risk for their children with T1DM.
- Family issues should be part of diabetes care guidelines.
- The social status of the father, a high level of employment and therefore sufficient financial resources seem to facilitate diabetes management.
- Children's metabolic control parameters are more favourable in mothers who work part-time or full-time than in mothers who stay at home in two-parent households.

Therefore, single parents, unemployed parents and special family forms need more professional psychosocial support [8, 22, 23, 28].
